# A cost-utility analysis of cervical cancer vaccination in preadolescent Canadian females

**DOI:** 10.1186/1471-2458-9-401

**Published:** 2009-10-31

**Authors:** Andrea M Anonychuk, Chris T Bauch, Maraki Fikre Merid, Georges Van Kriekinge, Nadia Demarteau

**Affiliations:** 1 Leslie Dan Faculty of Pharmacy, University of Toronto, Toronto, ON, Canada; 2 Statistics & Epidemiology, Medical Affairs, GlaxoSmithKline Canada, Mississauga, ON, Canada; 3 Department of Mathematics and Statistics, University of Guelph, Guelph, ON, Canada; 4 Department of Health Policy and Management Evaluation, University of Toronto, Toronto, ON, Canada; 5 Health Economics, GlaxoSmithKline Biologicals, Rixensart, Belgium

## Abstract

**Background:**

Despite the fact that approximately 70% of Canadian women undergo cervical cancer screening at least once every 3 years, approximately 1,300 women were diagnosed with cervical cancer and approximately 380 died from it in 2008. This study estimates the effectiveness and cost-effectiveness of vaccinating 12-year old Canadian females with an AS04-adjuvanted cervical cancer vaccine. The indirect effect of vaccination, via herd immunity, is also estimated.

**Methods:**

A 12-health-state 1-year-cycle Markov model was developed to estimate lifetime HPV related events for a cohort of 12-year old females. Annual transition probabilities between health-states were derived from published literature and Canadian population statistics. The model was calibrated using Canadian cancer statistics. From a healthcare perspective, the cost-effectiveness of introducing a vaccine with efficacy against HPV-16/18 and evidence of cross-protection against other oncogenic HPV types was evaluated in a population undergoing current screening practices. The base-case analysis included 70% screening coverage, 75% vaccination coverage, $135/dose for vaccine, and 3% discount rate on future costs and health effects. Conservative herd immunity effects were taken into account by estimated HPV incidence using a mathematical model parameterized by reported age-stratified sexual mixing data. Sensitivity analyses were performed to address parameter uncertainties.

**Results:**

Vaccinating 12-year old females (n = 100,000) was estimated to prevent between 390-633 undiscounted cervical cancer cases (reduction of 47%-77%) and 168-275 undiscounted deaths (48%-78%) over their lifetime, depending on whether or not herd immunity and cross-protection against other oncogenic HPV types were included. Vaccination was estimated to cost $18,672-$31,687 per QALY-gained, the lower range representing inclusion of cross-protective efficacy and herd immunity. The cost per QALY-gained was most sensitive to duration of vaccine protection, discount rate, and the correlation between probability of screening and probability of vaccination.

**Conclusion:**

In the context of current screening patterns, vaccination of 12-year old Canadian females with an ASO4-ajuvanted cervical cancer vaccine is estimated to significantly reduce cervical cancer and mortality, and is a cost-effective option. However, the economic attractiveness of vaccination is impacted by the vaccine's duration of protection and the discount rate used in the analysis.

## Background

Despite a large decline (>60%) in cervical cancer incidence and mortality since the implementation of screening in Canada in the 1960s, an estimated 1,300 women were diagnosed with cervical cancer and approximately 380 died in 2008 [1]. The guidelines for screening for cancer of the cervix in Canada state that all women aged 18 and over be screened, initially with two smears one year apart. If these smears are satisfactory then rescreening every three years is advised until the age of 69 [2]. The best national data currently available show 1 year participation rates do not vary greatly among provinces, ranging from 37% in British Columbia and Ontario to 44% in Nova Scotia. The 3-year participation rate in Canada is approximately 70% [3]. Despite screening programs, cervical cancer mortality has declined only marginally since 1990 [4]. Strong evidence points to specific high-risk human papillomavirus (HPV) types as the causative agent of the vast majority of cancerous and precancerous cervical lesions [5]. Specifically, HPV 16 and HPV 18 are implicated in approximately 70% of cervical cancers [3], and other high-risk HPV types including 31, 45, 33, 52 have been found to contribute an additional 14.5% [6]. Canadian data show that 85% of all HPV infection costs are related to screening and follow-up procedures, amounting to $250-$300 million annually [7].

In February 2007, the National Advisory Committee on Immunization (NACI) recommended vaccination of women between 9 and 13 years of age[4]. According to the Canadian Immunization Committee(CIC)-NACI HPV Working Group, the goal of an HPV immunization program in Canada is to decrease the morbidity and mortality of cervical cancer, its precursors and other HPV-related cancers in women through a combination of primary and secondary prevention strategies [4]. Two HPV vaccines exist at present, a quadrivalent HPV 6/11/16/18 (Gardasil^®^, Merck Frosst) currently available in Canada, and an HPV 16/18  AS04-adjuvanted vaccine (Cervarix™, GlaxoSmithKline) under regulatory review by Health Canada. Both vaccines are highly efficacious and safe [8-13]. Immunity against HPV is thought to be primarily type-specific [14-18], however there is some cross-protection between genetically related types. The HPV-16/18 AS04-adjuvanted vaccine is reported to provide high efficacy against CIN2+ associated with HPV-16/18 as well as non-vaccine oncogenic HPV individual types including HPV 45, HPV 31, HPV 33 [8,9]. Broad protection against CIN2+ infection with combined oncogenic, non-vaccine HPV types, suggesting that cross-protection might extend beyond types 45 and 31 [8,9]. In addition, substantial overall effect is reported in cohorts that are relevant to universal mass vaccination and catch-up programmes[8].

By reducing the amount of HPV transmission in the population, vaccination also reduces the risk of infection to unvaccinated individuals. This effect is termed herd immunity or herd protection[19,20], and its inclusion in the assessment of HPV vaccination generally results in lower cost-effectiveness ratios under certain common conditions [21-25]. Accounting for herd protection effects in a cost-utility analysis normally requires use of a dynamic model and thus abandonment of the spreadsheet-based cohort modeling framework often used for vaccination cost-effectiveness analysis[26].

Several studies have examined the cost-effectiveness of HPV vaccination in different countries. Though studies vary in model type, input data, and model assumptions, most of them have shown that HPV vaccination is likely to be cost-effective in pre-adolescent females[7,22-25,27-36]; however, the inclusion of male vaccination was generally not cost-effective[22-25,35] or in some cases less cost-effective and including catch-up programs was also less cost-effective[23,25]. Sensitivity analyses demonstrated that cost-effectiveness estimates of HPV vaccine are largely influenced by factors such as the discount rate, vaccine cost, vaccine efficacy, coverage, and duration of vaccine protection[7,22,25,27-31,33,34,36].

In this study, we estimate the effectiveness and cost-effectiveness of an AS04-adjuvanted cervical cancer vaccine in a hypothetical cohort of 12-year old females, in the context of current cervical cancer screening programs. We use a recently-developed methodology based on equations derived from dynamic models to conservatively account for herd immunity effects in the context of an existing spreadsheet-based Markov cohort model for HPV vaccination in Canadian women.

## Methods

### Model structure

**Figure 1:**
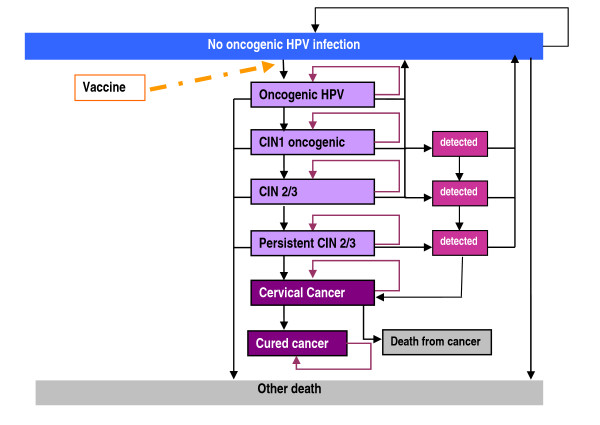
**Cohort model structure**. 12-disease state Markov process model with 12 month cycle, simulates natural history of HPV infection and cervical cancer. Transition rates vary by age, HPV type, screening, treatment, vaccination. Simple screening module. Detected: subjects with disease detected through screening have same pathways but different probabilities. CIN: Cervical Intraepithelial Neoplasia.

A simple and transparent Markov model was developed in Microsoft Excel that allows for rapid and flexible use with a primary focus on cervical cancer. This model was validated against a more complex model and adapted for its application to the Canadian setting[37,38]. Figure 1 displays the model schematics.

The Markov model replicates the natural history of cervical disease over the lifetime of the cohort in one-year cycles and in the following health states: Normal, HPV infection (high-risk, low-risk), Cervical Intraepithelial Neoplasia (CIN), Cervical Cancer, Cervical Cancer Death, and Other Death. Transition probabilities were age and HPV-type (high-risk, low-risk) specific and derived through the literature. Women in the model can progress and regress from states up to persistent CIN2,3 and thereafter may progress to cervical cancer. Women in the cancer state can either remain in cancer or move to a 'cured' cancer state and remain there over their lifetime. A simplified screening module is incorporated, with no follow-up testing after a negative cytology result, and colposcopy and biopsy with treatment after a positive cytology result. Regular (i.e., 70% women are screened every 3 years), irregular (i.e., 18% various screening patterns), and never screened (i.e. 12%) are taken into account[39]. Sensitivity for screening is incorporated to estimate the proportion of patients with their disease detected and treated. Natural immunity after HPV infection was not accounted for in the model. The number of sexual partners, age at first pregnancy, race and socioeconomic status are surrogate markers for infection with HPV[40] but were not included in the model.

### Calibration

The model was calibrated by manually manipulating input parameters to match the model output to observed epidemiological data while keeping transition probabilities within pre-determined ranges of plausibility. Age-specific cervical cancer incidence and mortality rates[41], as well as the overall number of cases and deaths estimated by the model were matched to observed data during this process. To validate the matching between the observed epidemiological data and model-predicted data, we conducted a chi-square test for significance at the level of p < 0.05.

### Vaccine efficacy

Vaccine efficacy is modelled by assuming it causes a reduction in the probability of acquiring HPV infection. The model uses an overall average vaccine effectiveness against all oncogenic types, computed as the average efficacy against HPV 16/18, and other non-vaccine oncogenic HPV types weighted by prevalence of cervical cancer attributed to each HPV-type[6]. Based on recently published data, we used 98% efficacy against HPV 16 and 18 infections and 37% for other non-vaccine oncogenic types[8,9]. Long-term data on vaccine efficacy is not currently available. Recently data shows strong and sustained antibody response for HPV 16 and HPV 18 up to 7.3 years post-vaccination [10,42] and mathematical models have predicted the long term vaccine duration to be at least 20 years[43]. Lifelong vaccine protection was assumed for the base-case analysis, and shorter duration times were tested in the sensitivity analysis. To prevent over-estimation of the vaccine benefit on the incidence of CIN events, CIN1 and CIN2,3 were thereafter added to take into account the difference between the proportion of HPV in CIN and cancer.

### Herd immunity

Herd protection effects were included within the cohort model through a pseudo-dynamic approximation based on the adaptation of a standard compartmental dynamic model, the Susceptible-Infectious-Recovered (SIR) model[44]. The model was calibrated using published estimates of HPV transmissibility[45,46]. The model equations were solved to determine the risk of infection to unvaccinated individuals in a population with a given level of vaccine coverage. This was combined with age-stratified data on sexual mixing patterns to determine the age-specific change in infection risk over time for those members of an aging cohort of women not successfully vaccinated under the vaccination program[24,47]. This change in infection risk was represented by a multiplicative factor rherd, representing the proportionate reduction in infection risk due to herd immunity effects. For instance, *r*_herd _= 0.5 means that susceptible individuals have their infection risk reduced by 50% due to vaccination in other members of the population). *r*_herd _can be set to 1 when herd immunity is not taken into account through this pseudo-dynamic approximation, i.e., when infection risk is assumed constant as under conventional Markov cohort models of vaccination. Details of the methodology appear in [47].

### Costs and Quality Adjusted Life Years (QALYs)

Total costs, total QALYs, number of cervical cancer cases, and number cervical cancer deaths were derived over the lifetime of one birth cohort of girls after assigning specific costs and QALY to each health state and cumulating those over the lifetime. Utilities for pre-cancer and cervical cancer were taken from the literature. The disutility of pre-cancer and cancer health states were subtracted from age-specific utility in the general population.

Data on costs were obtained from Canadian literature, though some of the data cited in the publications were derived from the US [7,48]. When necessary, costs were inflated to 2006 using the health and personal care component of the Consumer Price Index[49]. The cost of the vaccine was assumed to be $135 per dose. Cost of vaccine administration was not included in the base case analysis, though this may be represented in the upper bound of the vaccine cost in the sensitivity analysis. Cost for adverse events was not included, though HPV vaccine has been shown to have an excellent safety profile. Future costs and health effects were discounted at 3% and varied in sensitivity analyses.

Table 1 provides a summary of the input parameters and data sources. Base case estimates and ranges were determined using a combination of literature or best assumption. Data was Canadian-specific whenever possible.

**Table 1:** Summary of input parameters into health economic model

**Input parameters**	**Base Case Value**	**References**
***Vaccination***		

Efficacy HPV 16/18	98% (95% CI: 88.4-1000)	[8-11]

Efficacy non-vaccine oncogenic HPV types	37% (95% CI: 7.4-58.0)	[8,9]

Duration of vaccine protection	lifelong	Assumption

Coverage rate	75%	Assumption based on [50]

***Screening***		

Age and frequency	Age 18-69, 70% (once/3 years), 12% (never)	[39]

Cytology (Pap) test sensitivity	55.4%	[57]

Cytology (Pap) test specificity	96.8%	[57]

**Costs and utilities**		

***Vaccination costs***		

Vaccine per dose	$135.00	Assumption

***Screening Costs***		

Conventional cytology	$57	[7,48]

Colposcopy + Biopsy	$150	[7,48]

***Treatment Costs (annual)***		

CIN 1	$782	[7,48]

CIN 2/3	$1,353	[7,48]

Cervical cancer stage 1	$11,915	[7,48]

Cervical cancer stage 2	$18,851	[7,48]

Cervical cancer stage 3	$18,851	[7,48]

Cervical cancer stage 4	$25,759	[7,48]

***Utilities***		

Normal Population	0.94-0.89 (age-specific)	[58]

CIN lesion	0.96-0.995	[59,60]

Cervical cancer	0.73	[59,60]

Cured cancer	0.94	[61]

### Base case

Vaccination is evaluated in a hypothetical cohort of 100,000 12-year old Canadian girls. This age was chosen as the target group for vaccination because it represents women prior being at risk for HPV infection (i.e. before sexual debut). HPV vaccination rates differ across Canada by province. According to a Canadian report, HPV vaccination rates among school-based cohorts was 80% in Atlantic provinces, 85% in Newfoundland, 85-87% in Quebec, and 53% in Ontario [50]. Based on this variability we assumed a base-case rate of 75%. It was assumed that all adolescents would receive three doses of the vaccine and be fully immunized after 1 year. Vaccination is compared with the Canadian status quo for cervical cancer prevention, cytological (Pap) screening. For this analysis, we assume that current screening patterns will not change with implementation of vaccination. Four scenarios are included in base case analyses based on the inclusion or exclusion of herd protection and cross-protective efficacy. Sensitivity analysis was conducted on all four scenarios.

We conducted a cost-utility analysis from the perspective of the healthcare system (i.e., only direct costs to the healthcare system were taken into account), evaluating the incremental cost per QALY gained.

The incremental cost-utility ratio is calculated as:

(1)

where program A is current cervical cancer screening, and program B is cervical cancer vaccination plus current screening. As a threshold to decide whether the estimated incremental cost-effectiveness ratio (ICER) could be deemed cost-effective, we use the gross domestic product (GDP) per capita for Canada, $37,000 (2006 est.)[51].

### Sensitivity analysis

To deal with uncertainty in parameter values, sensitivity analyses were conducted to determine the impact of varying parameter values on the cost per QALY-gained. Several input parameters were varied within a pre-specified range of values based on published literature or best/worst case assumptions These included vaccine parameters (efficacy, duration of protection, coverage rate, age at vaccination), screening parameters (frequency), economic parameters (vaccine cost, screening costs, disease costs, discount rate), and quality of life parameters (utilities). We also tested a non-zero correlation between probability of screening and probability of vaccination, based on a Canadian survey of 500 adult women that found that contrary to their initial hypothesis, a higher proportion of females who had less regular screening tests were willing to recommend an HPV vaccine to their daughters/nieces [52]. For vaccine waning scenarios, we assume that vaccine efficacy does not wane until a specific time period after initial vaccination (e.g. 10 years, 30 years) after which it begins to wane linearly over a period of 5 years. Upper bound screening were varied by 50% from base case, as a recent review suggests the base case costs we used are conservative [48].

## Results

### Calibration

**Figure 2:**
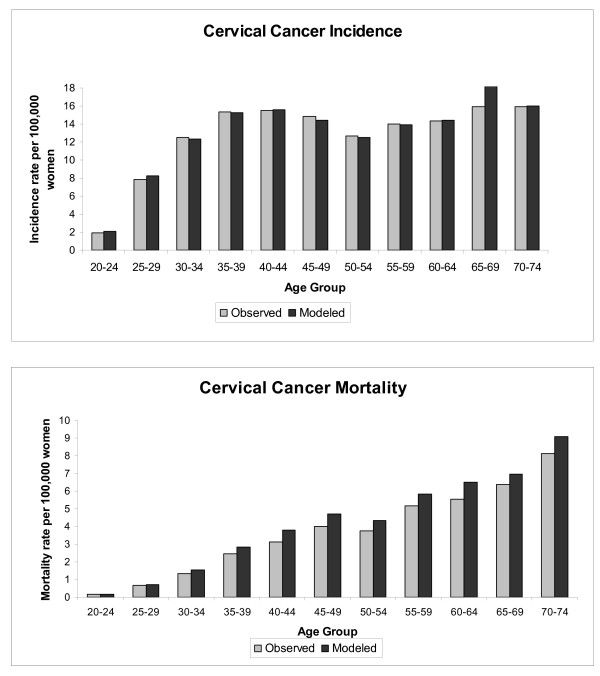
**Model Calibration: Observed vs. model predicted cases and deaths**. Note: The total number of observed vs. modeled cervical cancer cases and deaths were tested for differences using a Chisquare test for significance and evaluated at the p = 0.05 level. No statistically significant differences were found.

The model predicted and observed rates of cervical cancer incidence and deaths are compared in Figure 2. The modeled number of cumulative cervical cases over the lifetime of a 12 year old cohort is well matched to observed Canadian data (822 and 824, respectively). The model reflects, with a slight overestimation, the average Canadian cervical cancer death rates with a predicted cumulative 350 deaths compared with 319 observed over the lifetime of the cohort. The total number of observed vs. modeled cervical cancer cases and deaths were tested for differences using a Chi-square test for significance and evaluated at the p = 0.05 level. No statistically significant differences were found. Predictions are well matched to Canadian estimates when looking across five-year age groups as shown in figure 2.

In addition, the model predicts a cumulative 105,960 cases of CIN1 and 26,100 CIN2,3 compared with the 105,922 CIN1 and 25,294 CIN2,3 expected using the same epidemiologic data [41].

### Effectiveness of Vaccination

With a 75% vaccination rate, undiscounted base case scenario results estimate 390-457 cervical cancer cases and 168-196 cervical-related deaths would be prevented over the life-time a cohort of 100,000 girls vaccinated at 12 years of age compared with an unvaccinated cohort (Table 2). Discounted cases prevented ranged from 118-138 and 38-44 for cervical cancer and deaths, respectively. This represents at least a 47% reduction in cases and deaths. Taking into account herd protection effects, the estimated reduction in cervical cancer cases and deaths increases up to 77%-78%

**Table 2:** Undiscounted lifetime risk-reduction in burden of disease for bases case analyses, by duration of vaccine protection

	**No herd immunity**		**With herd immunity**	
	
	**HPV 16/18**	**HPV 16/18 + cross-protection**	**HPV 16/18**	**HPV 16/18 + cross-protection**
**Cancer Cases**				
No. cases no vaccination	822	822	822	822
No. cases with vaccination	432	365	369	189
No. cases prevented	390	457	453	633
% reduction	47.4%	55.6%	55.1%	77.0%
vaccine duration = 30 y	22.3%	26.2%	34.0%	61.6%
vaccine duration = 10 y	9.0%	9.0%	21.1%	51.8%

**Cancer Deaths**				
No. deaths no vaccination	351	351	351	351
No. deaths with vaccination	183	155	150	76
No. deaths prevented	168	196	201	275
% reduction	47.9%	55.8%	57.3%	78.3%
vaccine duration = 30 y	12.8%	15.1%	29.1%	57.7%
vaccine duration = 10 y	4.0%	4.0%	20.8%	51.4%

75% vaccination coverage.

Vaccine duration of protection: assumes initial vaccination efficacy lasts to 10 years, then wanes linearly over a 5 year period until 0% efficacy at year 16. 30 year scenario: efficacy lasts to 30 years, then wanes linearly for 5 year period until 0% efficacy at year 36.

Under a scenario where protection from the vaccine wanes after 10 years, the estimated reduction in undiscounted lifetime cancer cases and deaths is at least 9% and 4%, respectively (Table 2). With a vaccine with 30 years duration of protection the estimated reduction in undiscounted lifetime cancer cases and deaths is at least 22% and 13%, respectively. When herd protection effects are included, the estimated reductions in cervical cancer cases and deaths increase quite significantly to at least of 21% and 34% for 10 and 30 year duration of vaccine protection, respectively.

### Cost-utility of vaccination

Table 3 shows predicted cost-utility results in a vaccinated cohort under the base case scenarios compared with an unvaccinated cohort undergoing the same screening and treatment scenario. Compared with no vaccination, vaccinating 100,000 girls aged 12 years resulted in incremental QALYs gained of 0.0083-0.0095 per person and an incremental cost of $256 - $263 over the lifetime of the cohort. The resulting discounted cost per QALY gained for vaccination was in the range of $26,947 - $31,687 depending on whether or not cross-protection was included. The inclusion of herd immunity reduced the cost per QALY gained by 12% - 31%.

**Table 3:** Discounted cost, QALYs, and cost per QALY results for base case analyses, 3% discount, 75% vaccination coverage.

**Average cost and QALYs per person**	**Non-Vaccinated cohort**	**Vaccinated cohort**	**Incremental**	**Cost per QALY**
**No Herd Immunity**				

HPV 16/18				
Total Cost	$476	$739	$263	
Total QALYs	30.5015	30.5098	0.0083	$31,687

HPV 16/18 + cross protection				
Total Cost	$476	$732	$256	
Total QALYs	30.5015	30.5110	0.0095	$26,947

**With Herd Immunity**				

HPV 16/18				
Total Cost	$476	$735	$259	
Total QALYs	30.5015	30.5108	0.0093	$27,849

HPV 16/18 + cross protection				
Total Cost	$476	$715	$239	
Total QALYs	30.5015	30.5143	0.0128	$18,672

### Sensitivity Analysis

**Figure 3:**
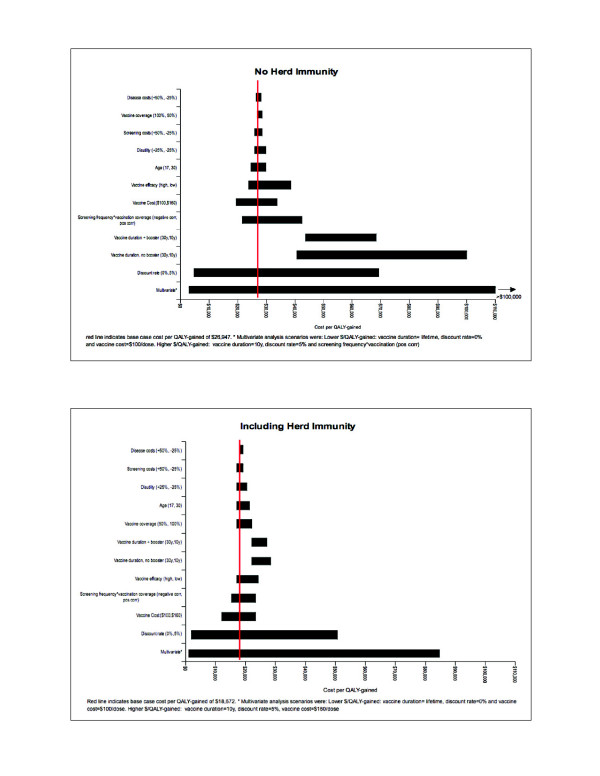
**Sensitivity analysis results for vaccination with HPV 16/18 + cross-protection, with and without herd immunity**.

Figure 3 shows results of the sensitivity analysis for vaccination with HPV 16/18 with cross-protective efficacy, with and without herd immunity. Results for HPV 16/18 only were similar, and therefore not shown here. HPV vaccination remained cost-effective for the most part (the cost per QALY gained remained below $37,000). The cost per QALY gained was less sensitive to changes in parameter values when herd immunity was included in the model.

The cost per QALY gained was generally not sensitive to changes in disease and screening costs, coverage, and age of vaccinated cohort. The cost per QALY gained was somewhat sensitive to changes in vaccine efficacy, and vaccine cost, though values still remained under the cost-effectiveness threshold.

Overall, the parameters that had the most impact on the cost per QALY-gained included the discount rate, duration of vaccine protection, and the correlation between probability of screening and probability of vaccination. Varying the discounting rates from 0% to 5% showed the greatest impact on the cost-utility ratio. The cost per QALY-gained varied from a low of $2,000 - $4,000 when a 0% discount rate was applied, to $50,000 - $69,000 when the discount rate was set to 5%. Given the lack of data on long-term duration of vaccine protection, we tested various waning immunity and booster scenarios. A scenario of linear waning of vaccine-derived immunity starting after 10 years of constant protection was the least effective strategy and resulted in cost per QALY-gained $68,000 and $100,000 with and without a booster, respectively. When herd protection effects are included, the cost per QALY-gained remains under the cost-effectiveness threshold. Results show that when protection is assumed to last 30 years, the cost per QALY does not change significantly when a booster is added, particularly when herd protection effects are included.

Our results indicate that if screening frequency and vaccination rates have a positive correlation (i.e. women who are infrequently or never screened are less likely to get vaccinated and vice versa), the cost per QALY-gained increases to $42,000. When herd immunity is taking into account, the cost per QALY-gained remains under the cost-effective threshold. If a negative correlation is assumed (i.e. women who are infrequently or never screened are more likely to get vaccinated and vice versa), the cost per QALY-gained decreases from our base case analysis, but does not go under $15,000 with or without inclusion of herd immunity.

Though no single parameter tested has resulted in a cost-savings situation, a multivariate sensitivity analysis that included varying only those parameters that had a positive impact on the cost per QALY-gained (i.e. lifelong vaccine protection, 0% discounting, lower vaccine cost), resulted in a very low cost per QALY gained of $1,000 - $2,000. Varying only the parameters that had a negative impact on the cost per QALY-gained (i.e. 10 year vaccine protection, 5% discounting, low vaccination rates among under-screened or never screened women) resulted in a cost per QALY-gained of $84,000 when herd protection was included to >$100,000 when herd immunity was not included.

## Discussion

The results from this cost-utility study suggest that within the context of current cervical cancer screening programs in Canada, cervical cancer vaccination of pre-adolescent/adolescent females is likely to be cost-effective from a healthcare perspective. These results were robust at the threshold of $37,000 per QALY-gained and under a wide range of assumptions and parameter uncertainties. Including herd immunity effects was estimated to increase base case vaccine effectiveness by approximately 8% - 23% and cost-effectiveness by 12% - 31%. Increases in vaccine effectiveness and cost-effectiveness are consistent with other economic analyses of HPV vaccination that have accounted for herd immunity effects [22-25,29]. Our base case cost-utility ratios are consistent with other Canadian analyses evaluating quadrivalent HPV female vaccination in a cohort model [7] and HPV 16/18 female vaccination in a dynamic model [53], showing cost per QALY-gained ranging from $21,000 - $33,115 per QALY-gained.

Our results indicate increased effectiveness and cost-effectiveness with a bivalent HPV 16/18 vaccine that confers cross-protection against other non-vaccine oncogenic HPV types (e.g. HPV 31, 33, 45) [8,9], though longer term clinical trial data is needed to understand vaccine efficacy for these individual HPV types. The inclusion of cross-protective efficacy led to greater disease and mortality reductions. Cross-protective efficacy alone without accounting for herd immunity resulted in an additional 67 cancer cases prevented and 28 deaths prevented.

Consistent with other cost-effectiveness studies of HPV vaccination [7,25,29,33,34,53], our results were sensitive to the discount rate and duration of vaccine protection. Because of the long latency between HPV vaccination and cervical cancer averted due to vaccination, we found that varying the discounting rates showed the greatest impact on the cost-utility ratio. According to much of the health economic literature, health effects should be discounted at the same rate as costs, however; some argue that health effects should be discounted at a lower rate than costs [54]. Others have suggested that discounting for vaccines should start from the time of risk reduction rather than the time of intervention [55,56].

Our results also suggested that duration of vaccine protection is important in the effectiveness and cost-effectiveness of vaccination. A vaccine where protection starts to wane before 30 years would result in less than a 26% reduction in cumulative cancer cases and 15% in deaths. A booster shot for older females may be warranted in this case, however vaccination compliance is often lower for adult programs than for school-based programs. For instance, compliance for adult Hepatitis A vaccination programs in Canada has been estimated to be 25% [26]. Under herd immunity, our results suggest that individuals who lose their vaccine-derived immunity later in life may still derive some indirect protection through herd immunity effects. However given the uncertainty and sensitivity of this parameter, surveillance of populations in coming years to determine the long-term duration of protection from HPV vaccines will be needed.

The cost-utility ratios are similar for various possible ages of vaccination between the ages 12-25. However, one should expect that vaccination at a younger age should generally result in lower cost-utility ratios, since more infection is thereby averted. In our results, cost-utility ratios may be similar across this age range because (a) HPV prevalence in Canada peaks at age 20-25, therefore vaccination before age 20 will prevent only a small number of additional events relative to vaccination at age 12, and (b) future cancer cases averted are discounted more heavily for vaccination at a younger age, which reduces estimated QALYs gains from a given expenditure on vaccination. This is confirmed by analyzing undiscounted results, in which the most favorable cost-effectiveness ratio is shown in the younger age cohort.

Our results also indicated that cost-effectiveness improves when women who are under-screened or never screened have higher vaccination rates than those women who are more frequent screeners. This occurs because the women who are vaccinated are those most at-risk for cervical cancer (i.e. under-screened or never screened), but fewer women in the total cohort are being vaccinated and therefore the total vaccine costs for cohort are reduced. However, the most effective strategy in terms of disease reduction occurs when a high coverage rate is to the entire cohort, irrespective of screening frequency, and this strategy remains below the cost-effectiveness threshold.

There are several limitations that should be noted. The model slightly overestimated the average Canadian cervical cancer death rate. It is possible that this could bias the study results in favour of the vaccine program. However, as noted earlier, our results are consistent with other economic analyses of cervical cancer vaccination in Canada and elsewhere. Varying levels of HPV infection in a population will have an impact on effectiveness and cost-effectiveness of vaccination programs and it is important to note that this was not taken into account here. Also, our base case value of vaccine cost was based on an assumption and did not include vaccine administration costs. Vaccine administration costs will vary depending on the vaccination strategy (e.g., new school-based program versus adding HPV vaccine to a pre-existing school-based program), therefore, it is important to look at the range of the cost-utility ratios in the sensitivity analysis under vaccination costs. Even in our upper range estimate of vaccine cost, the cost per QALY-gained was within the range of what is considered cost-effective.

We used a simple model to estimate the effectiveness and cost-effectiveness of cervical cancer vaccination in 12-year old females in Canada. This spreadsheet model requires fewer data and programming calculations which allows for rapid and flexible use. The value of such a simple model is that data requirements are more modest, which may suit its application to policy development in jurisdictions where data availability and/or quantity are limited. At the same time, this model has been validated against a more complex model and predicts similar cost-effectiveness [37]. We conservatively incorporated herd immunity effects in this cohort model of cervical cancer vaccination. The fullest and most accurate way to account for herd immunity effects is through a dynamic model, however, the approach described here allows us to approximate herd immunity on effects in the context of an existing spreadsheet-based cohort model under certain conditions. This may be useful as a means of preliminary incorporation of herd immunity effects preceding development of a full dynamic model.

This type of model can help inform policy until more complex models (i.e. sexual network transmission models) are available to answer and understand more in-depth policy questions. Some of the policy questions that will need to be explored are the long-term effectiveness and cost-effectiveness of current and possible modified cervical cancer screening policies (e.g. longer screening intervals and new technologies such as liquid-based cytology, HPV DNA testing) in the context of HPV vaccination in Canada.

## Conclusion

Using a recently-developed methodology to conservatively account for herd immunity effects in the context of an existing cohort model, our results suggest that immunization of pre-adolescent Canadian females with an ASO4-adjuvanted cervical cancer vaccine in 12 year old females is both effective at reducing cervical disease and deaths and is cost-effective when compared with cervical screening alone. However, the cost-effectiveness is highly influenced by the duration of protection of the vaccine and the discount rate used in the analysis.

## Abbreviations

CC: cervical cancer; CIN: cervical intraepithelial neoplasia; GDP: gross domestic product; HPV: human papillomavirus; ICER: incremental cost-effectiveness ratio; QALY: quality adjusted life year; SIR: susceptible-infectious-recovered.

## Competing interests

Funding for this project was provided by GlaxoSmithKline Canada. AA, ND, GVK are employed by GlaxoSmithKline. CTB and MFM have held various research contracts with GlaxoSmithKline Canada.

## Authors' contributions

AMA contributed to the development of the research question and methodology, analysis, interpretation of results, writing of the report, and project management. CTB contributed to the development of the research question, developed the methodology, and contributed to the interpretation of results and writing of the report. MFM and ND contributed to the methodology, analysis, interpretation of results. GVK contributed to the interpretation and writing of the report. All authors reviewed this paper.

## Pre-publication history

The pre-publication history for this paper can be accessed here:


